# Multilayered hybrid time-varying problem solving based on integrated-enhanced zeroing neural network for robust manipulator control

**DOI:** 10.1016/j.heliyon.2023.e20971

**Published:** 2023-10-17

**Authors:** Yansong Zhao, Jingjing Xiong

**Affiliations:** a Department of Foreign Languages, Xinyang Normal University, Xinyang 464000, China; b Xinyang Central Hospital, Xinyang 464000, China

**Keywords:** Manipulator control, Multi-task control, Multilayered hybrid time-varying problem, Integrated-enhanced zeroing neural dynamics, Robustness

## Abstract

Robustness is a significant research direction in manipulator control owing to their complicated and uncertain external environment, abrasion, and other factors. The ability to implement multitasking is also necessary for manipulator control because of the physical limitations and complex requirements. However, the existing research has mainly focused on the control of a single task and robustness analysis of single-task control. Although some research on multi-task control has been conducted recently, its robustness has not yet been studied. Because of the excellent performance of the integrated-enhanced zeroing neural network in terms of robustness for time-varying problem solving, it was employed in this study to solve robust multi-task control. First, the multi-task control was formulated as a two-layered time-varying problem, including nonlinear and hybrid linear equations describing the tracking task and additional tasks, respectively. Second, an integrated-enhanced zeroing neural network was employed for the multilayered time-varying problem solving and a robust multi-task control algorithm was obtained, which can suppress different types of noises. Theoretical analyses demonstrated its effectiveness in multitasking and superior robustness compared with conventional algorithms. Finally, simulation results verified the theoretical results.

## 1 Introduction

Redundant manipulator controls often appear in the industrial automation, medicine, and other fields [1], [2], [3]. For example, in [2], a redundant manipulator control was applied to industrial automation production by reducing complexity and computational effort. In [3], a controller based on state observer was developed for a 4-DOF manipulator applied to medical surgeries. The intelligence requirements for manipulator control algorithms are increasing. They require not only good real-time performance but also the capacity to effectively perform multiple tasks and good adaptability to the complex control environments [4], [5], [6]. Traditional control algorithms, such as PID and synovial control, perform well in ideal environments [7], [8], [9], [10]. For example, in [7], a fractional non-singular terminal sliding mode controller without manipulator dynamic information was presented. In [8], two algorithms were used to tune PID controllers to implement the control of a motor-actuated manipulator. In [9], an adaptively prescribed controller was developed for uncertain manipulators, which reduces the update frequency and avoids uninterrupted detection. However, these conventional control methods are deficient in terms of strict real-time performance, parameter selection, and robustness [11], [12], [13].

In recent years, a zeroing neural network (ZNN) method has been proposed and is gradually becoming widespread [14], [15]. Zhang et al. [14] proposed the concept of a ZNN for solving the time-varying Sylvester equation. In that study, the proposed method was termed a special recurrent neural network. Subsequently, different types of time-varying Sylvester equations were solved [15], [16]. In [15], the time-variant generalized Sylvester equation was investigated and solved using a ZNN method [14]. Furthermore, in [16], discrete-form Sylvester matrix problems were studied and solved using a ZNN. In addition, various strategies have been added to strengthen ZNNs [17], [18], [19], [20]. For example, in [17], an adaptive fuzzy control strategy was added to a ZNN for solving a time-variant QP problem. In [18], a ZNN assisted by a super-twisting algorithm was proposed to strengthen convergence and noise immunity. In [20], a time-varying parameter strategy was added to a ZNN resulting in a greater convergence performance.

The ZNN method treats and describes a problem from the perspective of time variation, dynamically solves the problem, and exhibits good real-time characteristics. This method also exhibits good real-time performance when applied to manipulator control [21], [22], [23], [24]. In [21], the manipulator control was modeled as a time-varying matrix inverse problem and solved by the obtained corresponding control algorithm. In [22], the manipulator control was modeled as linear equations system, and the proposed algorithm had a finite-time convergence property. In [23], the control problem was modeled as nonlinear equations, and a discrete-time control algorithm was proposed. The above control algorithms all have good real-time characteristics; however, problem modeling is relatively simple, can only complete the tracking task, cannot complete additional tasks, and is not robust in complex environments.

For the multi-task control problem, researchers have attempted to model the manipulator control problem as a multilayered time-varying problem. Different layers are used to describe different tasks, and simultaneous control of multiple tasks can be achieved when solving a multilayered time-varying problem. In [25], the control problem was modeled as a multilayered linear equation problem to realize the control of tracking and attitude. In [26], the control problem was modeled as a multilayered time-varying problem of equality and inequality to realize the simultaneous control of the tracking and joint angle limit. The multilayered structure modeling method solves the multi-task control problem well but is not robust in complex environments [27], [28].

In [29], integrated-enhanced ZNN was employed to solve a mathematical problem of hybrid linear equation system, and the corresponding solution is robust. Aiming at enhancing the robustness of multi-task algorithm, inspired by [29], a new solution method was used in this study. This method constructs the error function in integral form when solving multilayered hybrid time-varying problems and uses the integrated-enhanced ZNN to ensure that the final algorithm has strong robustness. In this study, theoretical analysis and numerical experiments were conducted for the robustness of algorithm in three environments: constant, linearly growing, and bounded random noise environments, and the advantages of the proposed algorithm were demonstrated by comparison with classical control algorithms.

In Section 2, the multi-task control of the manipulator is formulated. Section 3 solves the formulated time-varying problem by integrating enhanced zeroing neural dynamics, and presents theoretical analyses and conventional algorithms. Section 4 presents the simulation results that verify the theoretical results. Finally, Section 5 concludes the paper. The main contributions of this study are as follows:

1) A two-layered time-varying problem including nonlinear and hybrid linear equations and describing the basic tracking task and additional tasks is formulated for multi-task manipulator control.
2) A robust multi-task control algorithm is proposed using an integrated-enhanced ZNN, which can suppress different types of noises.
3) Theoretical proofs as well as numerical experiments guarantee the robustness of proposed algorithms.

## 2 Problem formulation of multi-task control

The multi-task control of a redundant manipulator is formulated as a multilayered hybrid problem here. First, according to the kinematic equations of robots, we obtained the following equation:

(1) $$f(\theta (t))=r_{a}(t).$$

Here, f(⋅) denotes the forward kinematic mapping function of the manipulator. θ(t) denotes the joint angle vector, which varies over time *t* during the control process. $r_{a}(t)$ denotes the actual trajectory of the end-effector. The basic tracking task in manipulator multi-task control refers to the actual trajectory $r_{a}(t)$ tracking a time-varying desired path, denoted as $r_{d}(t)$, in real time, i.e., $r_{a}(t)\to r_{d}(t)$. Therefore, combined with Equation (1), the first layer of the multilayered hybrid time-varying problem is defined as

(2) $$f(\theta (t))=r_{d}(t).$$

It is evident that if equation (2) is satisfied when the control process tends to be stable, the tracking control task can be completed.

While completing the tracking task, the control of redundant manipulators usually requires the completion of other tasks such as joint angle limit determined by hardware constraints, attitude control, and repetitive motion. In this study, the second layer of the multilayered hybrid time-varying problem was defined as

(3) $$A(t)\overset{˙}{\theta }(t)+B(t)\theta (t)+c(t)=0,$$

where A(t), B(t), and c(t) are user-defined time-varying matrices and vectors. The user can define different values of A(t), B(t) and c(t) as required, such that different additional tasks could be completed, such as the joint angle fixed. Finally, by combining (2) and (3), the multi-task control of the redundant manipulator is formulated as the following multilayered hybrid time-varying problem:

(4) $$\left\{ \begin{array}{cc} & f(\theta (t))=r_{d}(t)\\ & A(t)\overset{˙}{\theta }(t)+B(t)\theta (t)+c(t)=0. \end{array}\right.$$

## 3 Multi-task robust control algorithm

Problem (4) is solved based on an integrated enhanced ZNN, and a multi-task robust control algorithm is proposed in this part.

### 3.1 Development of algorithm

To solve problem (4) and obtain a robust control algorithm, the first layer of the problem was equivalently transformed into another form using the integrated-enhanced ZNN, and then merged with the second layer to obtain the final algorithm. The equivalent transformation process is as follows.

First, the time integral of the first layer of problem (4) is defined as error function:

(5) $$e_{1}(t)=\int_{0}^{t}(f(\theta (\tau ))-r_{d}(\tau ))d\tau .$$

By employing the ZNN design formula

$${\overset{˙}{e}}_{1}(t)=-\lambda e_{1}(t),$$

to zero out (5), we have

(6) $$f(\theta (t))-r_{d}(t)+\lambda \int_{0}^{t}(f(\theta (\tau ))-r_{d}(\tau ))d\tau =0.$$

Based on equation (6), we define the second error

(7) $$e_{2}(t)=f(\theta (t))-r_{d}(t)+\lambda \int_{0}^{t}(f(\theta (\tau ))-r_{d}(\tau ))d\tau .$$

Subsequently, using the ZNN design formula to zero out (7), we obtain the following equivalent equation:

(8) $$J(\theta (t))\overset{˙}{\theta }(t)-{\overset{˙}{r}}_{d}(t)+2\lambda (f(\theta (t))-r_{d}(t))+\lambda^{2}\int_{0}^{t}(f(\theta (\tau ))-r_{d}(\tau ))d\tau =0.$$

Combining equation (8) with the second layer of the multilayered hybrid time-varying problem (4) yields

(9) $$\begin{array}{c} \left[\begin{array}{c} J(\theta (t))\\ A(t) \end{array}\right]\overset{˙}{\theta }(t)+\left[\begin{array}{c} -{\overset{˙}{r}}_{d}(t)+2\lambda (f(\theta (t))-r_{d}(t))\\ B(t)\theta (t)+c(t) \end{array}\right]\\ =\left[\begin{array}{c} -\lambda^{2}\int_{0}^{t}(f(\theta (\tau ))-r_{d}(\tau ))d\tau \\ 0 \end{array}\right] \end{array}$$

We define $\vartheta (t)=\int_{0}^{t}(f(\theta (\tau ))-r_{d}(\tau ))d\tau$, and have

$$\overset{˙}{\vartheta }(t)=f(\theta (t))-r_{d}(t).$$

Equation (9) is rewritten as

(10) $$\left[\begin{array}{cc} I & 0\\ 0 & J(\theta (t))\\ 0 & A(t) \end{array}\right]\left[\begin{array}{c} \overset{˙}{\vartheta }(t)\\ \overset{˙}{\theta }(t) \end{array}\right]=\left[\begin{array}{c} f(\theta (t))-r_{d}(t)\\ {\overset{˙}{r}}_{d}(t)-2\lambda (f(\theta (t))-r_{d}(t))-\lambda^{2}\vartheta (t)\\ -B(t)\theta (t)-c(t) \end{array}\right]$$

which is a multi-task robust control algorithm for solving the multilayered hybrid time-varying problem (4).

A discussion regarding the proposed algorithm (10) is presented here. The time integral of the first layer of problem (4) is defined as the error function that leads to an integral algorithm. It is known that the integral operation generally enhances the robustness of the proposed algorithm. However, additional variable ϑ(t) is introduced into the algorithm because of the definition of the integral-formed error function. This leads to differential equations with larger dimensions compared with the classical ZNN method, which means that the proposed algorithm (10) has greater computational complexity. In addition, ODE solvers are employed to implement algorithm (10), and combined matrix [I,0;0,J(θ(t));0,A(t)] must be non-singular. In addition, only the integral-formed error function for the first layer was defined, and the second layer was directly added to the algorithm. Thus, the effectiveness of the proposed algorithm may not be guaranteed when noise exists in the second layer. This is a direction for future research. Beside, if integer derivatives are replaced by fractional derivatives during the development of algorithm, it may be still effective for manipulator control. It is an interesting further research direction [30].

### 3.2 Theoretical analyses

Two theorems about algorithm (10) are shown as follows.

Theorem 1 *Starting from an effective initial state for the manipulator, the multi-task control algorithm*(10)*is exponentially convergent, and the tracking error and the error for additional task tend to zero, that is,*

$$\underset{t\to \infty }{\lim}||f(\theta (t))-r_{d}(t)||+||A(t)\overset{˙}{\theta }(t)+B(t)\theta (t)+c(t)||=0.$$

Proof. According to the multi-task control algorithm (10), Equation (10) is equivalent to Equation (9), which can be divided into two parts: Equation (8) and Equation (3), and the latter can make the error of additional tasks tend to zero, that is,

$$\underset{t\to \infty }{\lim}||A(t)\overset{˙}{\theta }(t)+B(t)\theta (t)+c(t)||=0.$$

Define the tracking error function $e(t)=f(\theta (t))-r_{d}(t)$, and we have

$$\overset{˙}{e}(t)+2\lambda e(t)+\lambda^{2}\int_{0}^{t}e(\tau )d\tau =0.$$

By elementalization, we have

$${\overset{˙}{e}}_{i}(t)+2\lambda e_{i}(t)+\lambda^{2}\int_{0}^{t}e_{i}(\tau )d\tau =0.$$

The derivative of the above equation leads to the following homogeneous ordinary differential equation:

$${\overset{¨}{e}}_{i}(t)+2\lambda {\overset{˙}{e}}_{i}(t)+\lambda^{2}e_{i}(t)=0.$$

By solving the above equation, we have

$$\underset{t\to \infty }{\lim}\;e_{i}(t)=0.$$

Besides, $e_{i}(t)$ exponentially converges to zero. Finally, we have

$$\underset{t\to \infty }{\lim}||f(\theta (t))-r_{d}(t)||=0$$

and error function exponentially converges to zero. The proof is completed.

Theorem 2 *The multi-task control algorithm*(10)*is robust in various types of noisy environments. The error of algorithm*(10)*with a constant noise exponent converges to 0. In the presence of linearly growing unbounded or bounded random noise, the error of algorithm*(10)*is bounded, and the error bound decreases as the parameter λ increases.*

Proof. To analyze the robustness of multi-task control algorithm (10), noise notation n(t) is added to the algorithm. Based on the proof of Theorem 1, we have

$${\overset{˙}{e}}_{i}(t)+2\lambda e_{i}(t)+\lambda^{2}\int_{0}^{t}e_{i}(\tau )d\tau =n_{i}(t),$$

where $n_{i}(t)$ denotes any element of noise vector. The proof is divided into three parts: constant noise, linearly growing unbounded noise, and bounded random noise.

1) Constant noise. Considering constant noise, we have n(t)=n, and

$${\overset{˙}{e}}_{i}(t)+2\lambda e_{i}(t)+\lambda^{2}\int_{0}^{t}e_{i}(\tau )d\tau =n_{i}.$$

The derivative of the above equation leads to the following second-order homogeneous ordinary differential equation:

$${\overset{¨}{e}}_{i}(t)+2\lambda {\overset{˙}{e}}_{i}(t)+\lambda^{2}e_{i}(t)=0.$$

By solving this differential equation, $e_{i}(t)$ converges exponentially to zero. Finally, we obtain

$$\underset{t\to \infty }{\lim}||f(\theta (t))-r_{d}(t)||=0,$$

and error function converges exponentially to zero.

2) Linearly growing unbounded noise. When considering linearly growing unbounded noise, we have n(t)=nt, and

$${\overset{˙}{e}}_{i}(t)+2\lambda e_{i}(t)+\lambda^{2}\int_{0}^{t}e_{i}(\tau )d\tau =n_{i}t.$$

The derivative of the above equation leads to the following second order homogeneous ordinary differential equation:

$${\overset{¨}{e}}_{i}(t)+2\lambda {\overset{˙}{e}}_{i}(t)+\lambda^{2}e_{i}(t)=n_{i}.$$

By solving this differential equation, we have

$$e_{i}(t)=c_{1}e^{\theta t}+c_{1}te^{\theta t}+\frac{n_{i}}{\lambda^{2}}.$$

Therefore, in a linearly growing unbounded noise environment, the error is bounded and the error bound decreases as the parameter increases.

3) Bounded random noise. When considering bounded random noise, we have

$${\overset{˙}{e}}_{i}(t)+2\lambda e_{i}(t)+\lambda^{2}\int_{0}^{t}e_{i}(\tau )d\tau =n_{i}(t).$$

Based on the proof of Lemma 3 in [21], the error is also bounded and it decreases as the parameter increases. The proof is completed.

### 3.3 Comparisons with conventional algorithms

Conventional algorithms include a single-task control algorithm based on a single-layered problem formulation and a non-integral multi-task control algorithm. Here, we introduce the development processes of the two conventional algorithms and discuss the differences between our algorithm and the conventional algorithms.

When we develop a single-task control algorithm, only the tracking task is formulated:

$$f(\theta (t))\to r_{d}(t).$$

We define

$$e(t)=f(\theta (t))-r_{d}(t).$$

ZNN design formula

$$\overset{˙}{e}(t)=-\lambda e(t)$$

is employed, and the single-task control algorithm is then obtained as

(11) $$J(\theta (t))\overset{˙}{\theta }(t)={\overset{˙}{r}}_{d}(t)-\lambda (f(\theta (t))-r_{d}(t)).$$

When we develop a non-integral multi-task control algorithm, the multi-task problem formulation (4) is considered. Based on the single-task control algorithm (11), the first layer of (4) is equivalently converted as

$$J(\theta (t))\overset{˙}{\theta }(t)={\overset{˙}{r}}_{d}(t)-\lambda (f(\theta (t))-r_{d}(t)).$$

Combining this equation with the second layer yields the non-integral multi-task control algorithm as follows:

(12) $$\left[\begin{array}{c} J(\theta (t))\\ A(t) \end{array}\right]\overset{˙}{\theta }(t)=\left[\begin{array}{c} {\overset{˙}{r}}_{d}(t)-\lambda (f(\theta (t))-r_{d}(t))\\ c(t)-B(t)\theta (t) \end{array}\right]$$

There are some differences between our algorithm (10) and conventional algorithms. Conventional algorithms (11) and (12) have simpler structures and require fewer calculations than our algorithm (10). However, matrix inversion, which is the most time-consuming step, is inevitable in the implementation of the three algorithms. In addition, the conventional algorithm (11) can only complete the basic tracking task, and cannot suppress any types of noise, such as constant, random bounded or linearly growing noise. Specifically, when we consider noise n(t) in the conventional algorithms (11) and (12), they are expressed as

$$J(\theta (t))\overset{˙}{\theta }(t)={\overset{˙}{r}}_{d}(t)-\lambda (f(\theta (t))-r_{d}(t))+n(t),$$

which is exactly

$$\overset{˙}{e}(t)=-\lambda e(t)+n(t).$$

Then, we have

$${\overset{˙}{e}}_{i}(t)=-\lambda e_{i}(t)+n_{i}(t).$$

When considering constant noise, i.e., $n_{i}(t)=n$, we have

$${\overset{˙}{e}}_{i}(t)=-\lambda e_{i}(t)+n.$$

By solving this differential equation, we have

$${\overset{˙}{e}}_{i}(t)=-\lambda \exp(-\lambda t)+n.$$

It is observed that the tracking error cannot converge to zero as it is affected by noise *n*. When consider random bounded noise, we have

$${\overset{˙}{e}}_{i}(t)=-\lambda e_{i}(t)+n_{i}(t).$$

Solving this differential equation prevents the tracking error from converging to zero. When considering linearly growing noise, the tracking error does not converge in a similar manner.

## 4 Simulation verifications

Many simulation results based on the planar 6-link manipulator and PUMA560 manipulator are illustrated, including the comparative results of our algorithm (10), conventional algorithm (11), which is based on a single-layer problem formulation, and conventional algorithm (12), which is based on a ZNN without integration. Based on the theoretical analyses in the above section, we know that our algorithm (10) not only completes multi-tasks but also suppresses different types of noises, including constant, linear growing and random bounded noises. However, the conventional algorithm (11) cannot complete multi-tasks and cannot suppress any types of noises. The conventional algorithm (12) cannot suppress any types of noises although it can complete multi-tasks.

### 4.1 Planar 6-link manipulator

Numerical experimental results based on a planner 6-link manipulator are shown here. The task duration is set as 20 s; The initial state of joint angle of manipulator is [π/5,−3π/5, ${-\pi /4,\pi /6,\pi /3,-\pi /3]}^{T}$; Additional task is to keep the attitude of end-effector fixed, and thus, we define matrices $A(t)={\left[1,1,...,1\right]}^{T}$, $B(t)={\left[0,0,...,0\right]}^{T}$ and c(t)=0, which means $\theta_{1}+\theta_{2}+...+\theta_{6}$ is fixed. The desired path is a crescent, and its parametric equation is as follows:

$$\left[\begin{array}{c} X\\ Y \end{array}\right]=\left[\begin{array}{c} 0.25\cos(0.1\pi t)+0.25\cos(0.2\pi t)\\ 0.25\sin(0.1\pi t) \end{array}\right].$$

Note that this desired path is not pre-set and future path information is unknown at current instant. We use “ODE45” in MATLAB to execute the algorithms.

**Figure 1:**
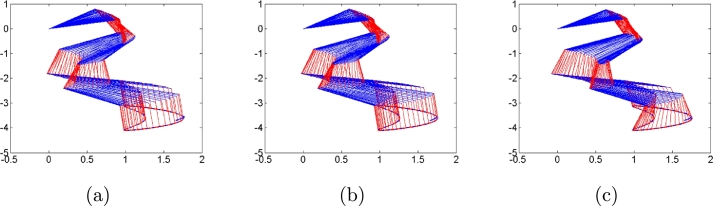
Robot trajectories generated by (a) algorithm (10), (b) algorithm (11) and (c) algorithm (12) when no noises are considered.

**Figure 2:**
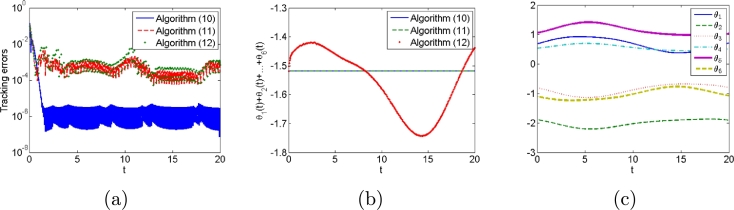
(a) Tracking errors and (b)states of additional task generated by algorithms (10), (11) and (12), and (c) all control input states *θ*(*t*) when no noises are considered.

First, we do not consider any types of noises in the control process to compare algorithms (10), (11) and (12). Simulation results are shown in Figure 1, Figure 2. Fig. 1 shows robot trajectories generated by algorithms (10), (11) and (12) when no noises are considered. It is observed that all algorithms can complete basic tracking task. However, Fig. 2 shows tracking errors and states of additional task generated by algorithms (10), (11) and (12), which illustrates that algorithms (10) and (11) complete the additional task successfully, whereas algorithm (12) fails to complete the additional task. The simulation results agree with the theoretical results. Besides, the trajectories of controller inputs θ(t) are shown in Fig. 2(c), from which their boundedness is demonstrated.

**Figure 3:**
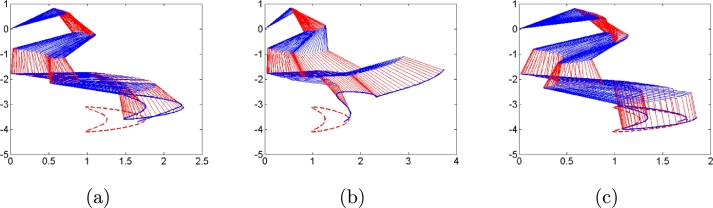
Robot trajectories generated by conventional algorithm (11) when (a) constant noises, (b) linear growing noises and (c) random bounded noises are considered.

**Figure 4:**
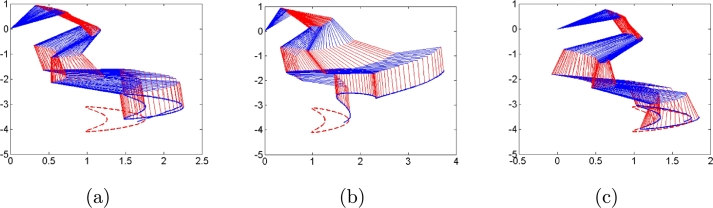
Robot trajectories generated by conventional algorithm (12) when (a) constant noises, (b) linear growing noises and (c) random bounded noises are considered.

**Figure 5:**
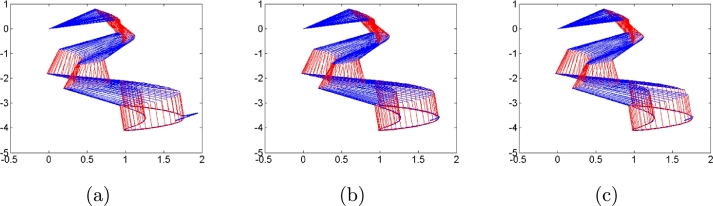
Robot trajectories generated by our algorithm (10) when (a) constant noises, (b) linear growing noises and (c) random bounded noises are considered.

**Figure 6:**
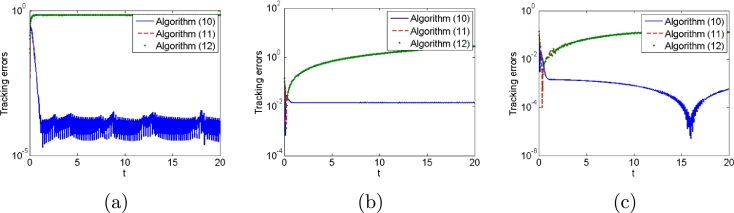
Tracking errors generated by algorithms (10), (11) and (12) when (a) constant noises, (b) linear growing noises and (c) random bounded noises are considered.

**Figure 7:**
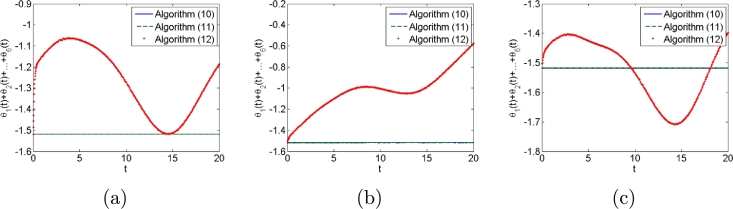
Additional task states generated by algorithms (10), (11) and (12) when (a) constant noises, (b) linear growing noises and (c) random bounded noises are considered.

Second, we consider three types of noises, i.e., constant, linearly growing and random bounded noises to illustrate the good performances of algorithms (10) compared with conventional algorithms (11) and (12). The three types of noises are arbitrarily set according to the noise characteristics as follows: All elements of constant noise vector are set as 5; All elements of linear growing noise vector are $n_{i}(t)=t$; All elements of random bounded noise art set as sin⁡(0.1t). Simulation results are shown in Figure 3, Figure 4, Figure 5, Figure 6, Figure 7. Fig. 3 shows the trajectories of manipulator as well as the trajectories of end-effector generated by conventional algorithm (11) in the presence of three types of noises. It agrees with theoretical result that algorithm (11) fails to complete basic tracking task under the three types of noise environments. Fig. 4 shows the trajectories of manipulator as well as the trajectories of end-effector generated by conventional algorithm (12) in the presence of three types of noises. Similar to algorithm (11), it agrees with theoretical result that algorithm (12) fails to complete basic tracking task. Fig. 5 shows the trajectories of manipulator as well as the trajectories of end-effector generated by conventional algorithm (10) in the presence of three types of noises. It agrees with theoretical result that algorithm (10) completes basic tracking task under the three types of noise environments. The end-effector trajectories quickly track the desired path, and then vary as the desired path in real time. Figure 6, Figure 7 show tracking errors and additional task states generated by algorithms (10), (11) and (12) when constant, linearly growing and random bounded noises are considered. It is observed that the tracking errors generated by algorithm (10) are much lower than those of the other two algorithms from Fig. 6. The sum of joint angle values $\theta_{1}(t)+\theta_{2}(t)+...+\theta_{6}(t)$ remains unchanged during the control process for algorithms (10) and (12) from Fig. 7 whereas algorithm (11) does not. It agrees with theoretical result that algorithms (10) and (12) can complete multi tasks whereas algorithm (11) does not. Furthermore, the actual computational time of algorithm (10) in the presence of different types of noises is around 0.43 s, which is much less the task duration 20 s. Besides, the actual computational time of conventional algorithms (11) and (12) in the presence of different types of noises are around 0.22 s and 0.19 s. The simulation results are consistent with theoretical discussions, i.e., computational complexity of our algorithm is slightly larger than that of conventional algorithms (11) and (12).

**Figure 8:**
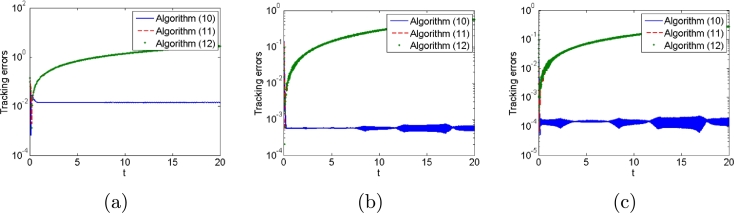
Tracking errors generated by algorithms (10), (11) and (12) with different values of parameter *λ* (a) *λ* = 10, (b) *λ* = 50 and (c) *λ* = 100.

**Figure 9:**
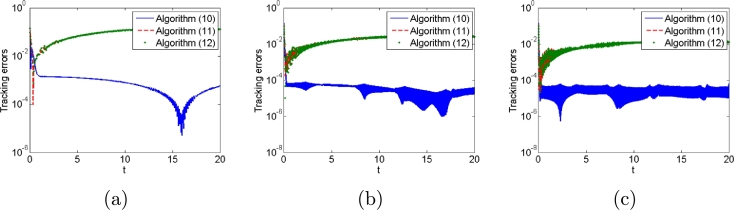
Tracking errors generated by algorithms (10), (11) and (12) with different values of parameter *λ* (a) *λ* = 10, (b) *λ* = 50 and (c) *λ* = 100.

Third, we illustrate the effect of parameter *λ* to the algorithms. For this purpose, we use different values of parameter *λ*, i.e., λ=10,50,100 in the presence of different types of noises. Simulation results are shown in Figure 8, Figure 9. From these two figures, when the values of parameter *λ* increase, the tracking error decrease, which also agrees with the theoretical results.

### 4.2 PUMA560 manipulator

**Figure 10:**
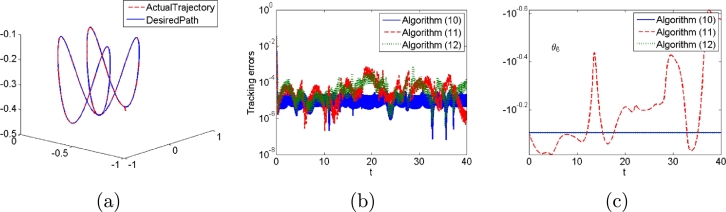
End-effector trajectories, tracking errors and additional task states generated by algorithms (10), (11) and (12) for PUMA560 manipulator when no noises are considered. (a) end-effector trajectories generated by algorithm (10), (b) tracking errors, (c) additional task states.

Numerical experimental results based on PUMA560 manipulator are presented herein. In these simulations, task duration is set as 40 s; Initial state of joint angle of manipulator is set as ${\left[0,-\pi /2,0,\pi /2,\pi /2,-\pi /4\right]}^{T}$; Additional task is to fix the 6th joint angle, and thus, we define matrices $A(t)={\left[0,...,0,1\right]}^{T}$, $B(t)={\left[0,0,...,0\right]}^{T}$ and c(t)=0, which means $\theta_{6}$ is fixed. The desired path is shown in Fig. 10(a).

First, we consider no noises in the control process to compare algorithms (10), (11) and (12). The results are shown in Fig. 10. Tracking errors all quickly converge for the three algorithms, which means that the tracking task is completed for them from Fig. 10(b). The values of $\theta_{6}$ are fix for algorithms (10) and (12) whereas that of algorithm (11) does not, which means that algorithms (10) and (12) complete additional task whereas algorithm (11) does not from Fig. 10(c).

**Figure 11:**
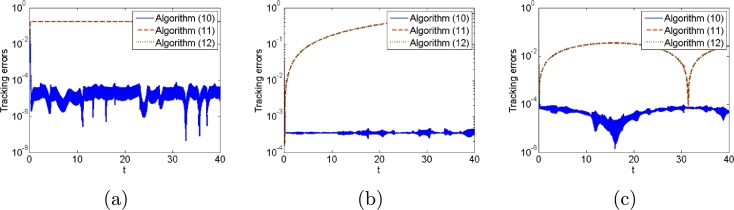
Tracking errors generated by algorithms (10), (11) and (12) for PUMA560 manipulator when (a) constant noises, (b) linear growing noises and (c) random bounded noises are considered.

**Figure 12:**
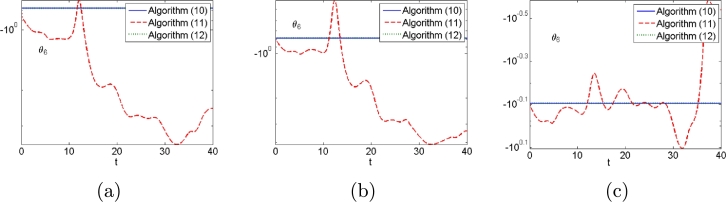
Additional task states generated by algorithms (10), (11) and (12) for PUMA560 manipulator when (a) constant noises, (b) linear growing noises and (c) random bounded noises are considered.

Second, we consider different types of noises in the control process. The results are shown in Figure 11, Figure 12. Tracking errors of algorithm (10) are always convergent with different types of noises whereas those of algorithms (11) and (12) do not from Fig. 11. Algorithms (10) and (12) with different types of noises complete additional task whereas algorithm (11) does not from Fig. 12.

## 5 Conclusion

In this study, multi-task control of manipulator has been formulated as two layered time-varying problem in this work. This type of formulation describes not only tracking task, but also additional tasks. The multilayered time-varying problem has been solved by integrated-enhanced ZNN, and the robust multi-task control algorithm has been obtained. Note that the proposed algorithm can suppress different types of noises, such as constant, linear growing and random bounded noises whereas conventional algorithms cannot suppress these noises. Theoretical analyses demonstrate the effectiveness in the aspect of multi tasks and robustness. Finally, simulation results have been illustrated to verify theoretical results. Besides, the effectiveness of proposed algorithm may not be guaranteed when noises exist in the second layer, which is a future research direction of this work.

## Funding statement

This project was supported by Henan Graduate Education Quality Course Project (No. YJS2022KC34); and Key Project of Teacher Education Curriculum Reform in Henan Province (No. 2022-JSJYZD-018).

## Additional information

No additional information is available for this paper.

## CRediT authorship contribution statement

**Yansong Zhao:** Methodology, Writing – original draft. **Jingjing Xiong:** Methodology, Writing – review & editing.

## Declaration of Competing Interest

The authors declare that they have no known competing financial interests or personal relationships that could have appeared to influence the work reported in this paper.

## Data Availability

Data will be made available on request.
